# Aberrant angiogenic signaling in HCC: therapeutic targeting and drug resistance

**DOI:** 10.3389/fonc.2025.1595195

**Published:** 2025-06-18

**Authors:** Hongtao Zhang, Zhaoming Yang, Zhengwu Jiang, Zhijian Zhao, Xun Chen, Jian Wan, Yukun Li

**Affiliations:** ^1^ Jishou University Zhuzhou Clinical College, Medical College, Jishou University, Zhuzhou, Hunan, China; ^2^ Second Department of Hepatobiliary Pancreatic and Splenic Surgery, Medical Center of Digestive Disease, Zhuzhou Hospital Affiliated to Xiangya School of Medicine, Central South University, Zhuzhou, Hunan, China; ^3^ Department of Assisted Reproductive Center, Zhuzhou Hospital Affiliated to Xiangya School of Medicine, Central South University, Zhuzhou, Hunan, China

**Keywords:** hepatocellular carcinoma, angiogenesis, inhibitor, pathways, drug resistance

## Abstract

Liver cancer ranks as the sixth most prevalent malignancy globally, with Hepatocellular Carcinoma (HCC) constituting the predominant subtype, thereby imposing a significant burden on public health and presenting limited therapeutic options. Despite ongoing efforts to innovate treatment modalities, anti-angiogenesis therapy continues to be the primary strategy for managing HCC. Angiogenesis is a pivotal process within the tumor microenvironment, characterized by the formation of new blood vessels that provide essential nutrients and oxygen to proliferating tumors, thereby facilitating their growth and potential metastasis. Numerous angiogenic signaling pathways become dysregulated during this process. Targeting these aberrant pathways can yield significant therapeutic benefits for patients and may even reverse drug resistance. However, these signaling pathways frequently demonstrate intricate crosstalk and interconnections. Elucidating these interactions could represent a crucial strategy for advancing the treatment of HCC. This review provides both mechanistic insights into angiogenic network plasticity and translational strategies to overcome therapeutic bottlenecks in HCC management.

## 1 Introduction

Liver cancer ranks as the sixth most prevalent malignancy globally and constitutes the third leading cause of cancer-related mortality. HCC represents approximately 90% of primary liver cancer cases, thereby exerting considerable pressure on public health (1). While surgical intervention, conventional radiotherapy, and chemotherapy offer therapeutic options for HCC, these traditional modalities exhibit limited efficacy in patients with advanced and incurable HCC. Furthermore, approximately 40-70% of patients experience disease recurrence within five years following surgical resection (2). Consequently, identifying novel strategies for the treatment of HCC is of paramount importance. The 2024 EASL Clinical Practice Guidelines highlight that first-line targeted therapies for HCC predominantly utilize multi-tyrosine kinase inhibitors (multi-TKIs), which exert anti-angiogenic effects primarily through VEGF receptor blockade while demonstrating ancillary activity against FGFR, TIE2, and MET pathways (3). Beyond these established therapeutic targets, accumulating evidence from preclinical studies and comprehensive reviews reveals that the NOTCH (4), Wnt/β-catenin (5), PI3K/AKT (6), pathway are aberrantly activated in HCC. These evolutionarily conserved pathways have been mechanistically linked to multiple oncogenic processes, exhibiting profound implications for tumor angiogenesis, proliferation, metastasis, and drug resistance. This review aims to this review aims to systematically evaluate the molecular mechanisms underlying aberrant activation of key angiogenic signaling pathways (NOTCH, Wnt/β-catenin, Ang/Tie, FGF, HGF, VEGF, and PI3K/AKT) in HCC progression and therapeutic resistance. We will critically analyze emerging preclinical and clinical evidence regarding pathway crosstalk within the tumor microenvironment, with particular focus on their synergistic contributions to angiogenesis, immune evasion, and acquired drug resistance. Furthermore, this review will assess current therapeutic strategies targeting these pathways, including combination approaches with immune checkpoint inhibitors, while highlighting persisting challenges in clinical translation and proposing rational polytherapy frameworks to overcome compensatory signaling adaptation.

## 2 Notch pathway

**Figure 1:**
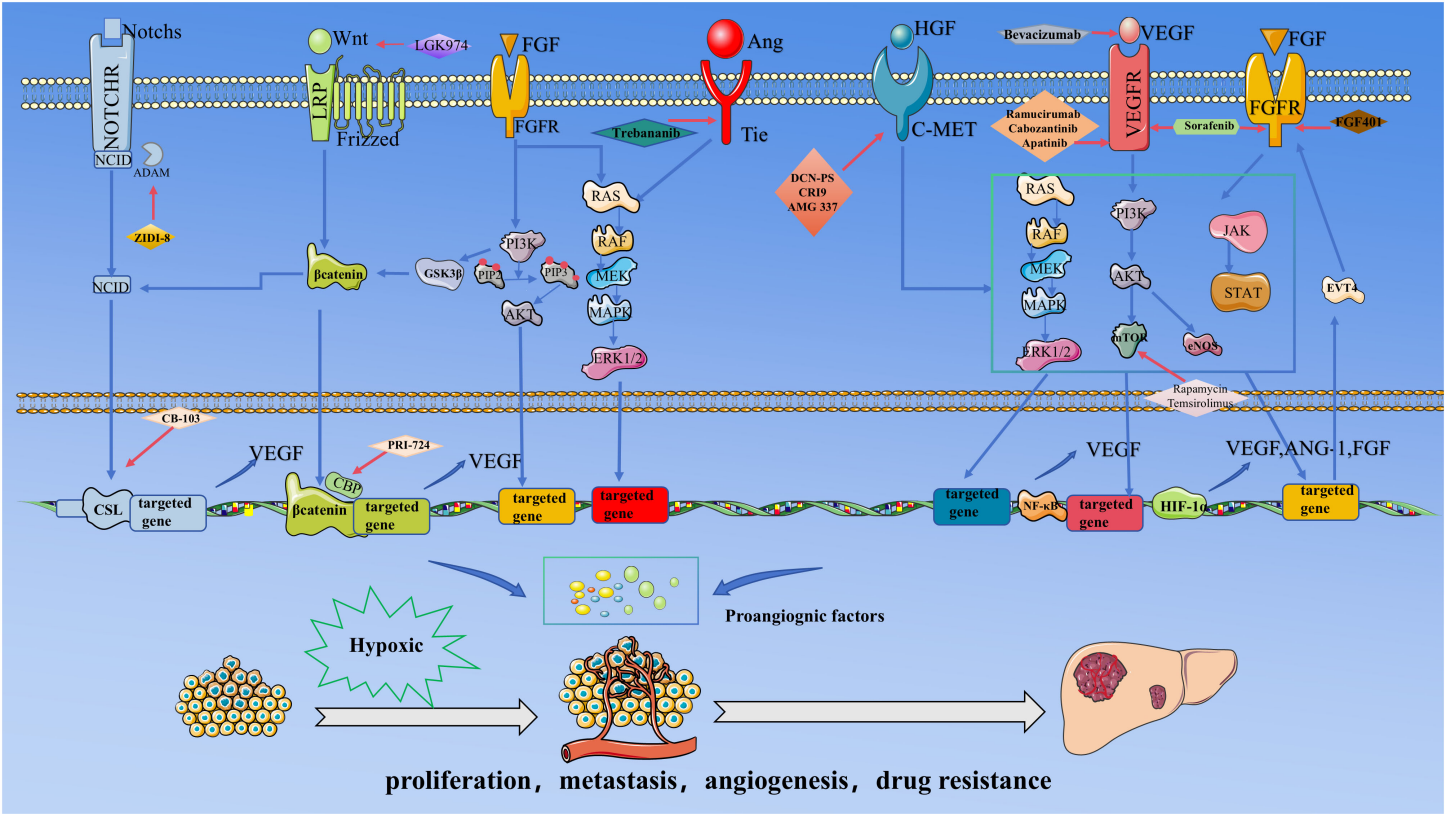
.This figure delineates the core signaling network and targeted therapeutic strategies governing angiogenesis in HCC under hypoxic tumor microenvironment. During HCC progression, rapid tumor cell proliferation induces localized hypoxia, stabilizing HIF-1α and augmenting its transcriptional activity. HIF-1α directly drives the expression of pro-angiogenic factors, including VEGF, FGF, and Ang-1. Concurrently, pathways such as Notch, Wnt/β-catenin, Ang/Tie, and PI3K/AKT are hyperactivated, collectively promoting pathological angiogenesis. The resulting neovasculature facilitates oxygen and nutrient delivery, further accelerating tumor proliferation, metastasis, and drug resistance.Critical inter-pathway cross-talk underpins this process: The activation of the NICD is mechanistically linked to Wnt/β-catenin signaling, wherein NICD synergizes with β-catenin to amplify transcriptional output; FGF signaling stabilizes β-catenin via GSK-3β phosphorylation, enhancing Wnt pathway activity; and the PI3K/AKT-mTOR axis stabilizes HIF-1α, establishing a Positive feedback loop with VEGF to sustain angiogenic signaling.Therapeutic strategies target multiple regulatory nodes: Bevacizumab neutralizes VEGF ligands to inhibit endothelial activation; Sorafenib suppresses VEGFR and FGFR kinase activity; DZW-301 attenuates PI3K downstream signaling; ZIDI-8 inhibited γ-secretase inhibitor and CB-310 disrupted NICD nuclear translocation, thereby inhibiting notch signalling.

The Notch signaling genes are highly conserved within the human body and play a critical role in regulating cell proliferation and differentiation, embryonic development, and tissue homeostasis, among other processes. This signaling pathway comprises four transmembrane receptors, Notch1 through Notch4, which engage with five transmembrane ligands: Jagged1, Jagged2, Dll1, Dll3, and Dll4. Upon interaction between a Notch ligand and its corresponding receptor, the extracellular domain of the Notch receptor undergoes proteolytic cleavage. This is followed by additional cleavage mediated by γ-secretase, resulting in the release of the Notch intracellular domain (NICD) from the cell membrane. The NICD is subsequently translocated into the nucleus, where it interacts with the CSL transcription factor complex, thereby initiating the transcription of target genes. This process ultimately influences a range of biological outcomes, including cell proliferation and differentiation (**Figure 1**) (7).

The Notch signaling pathway is markedly upregulated in HCC and plays a significant role in the initiation and progression of tumorigenesis (8). Consequently, conducting comprehensive research on the mechanisms of the Notch signaling pathway in HCC and developing specific inhibitors targeting this pathway is of paramount importance (**Table 1**). Experimental models demonstrate that pharmacological modulation of Notch activity through γ-secretase inhibitors (e.g., DAPT) can restore cellular homeostasis by counterbalancing EGFL8 deficiency-induced pathway hyperactivity (9). In addition, natural compounds targeting Notch signaling pathways exhibit multifaceted therapeutic effects. Application of matrine demonstrates dual hepatoprotective functions: while attenuating Notch-driven oncogenic signaling, this alkaloid promotes hepatic oval cell differentiation into functional hepatocytes, ameliorating histopathological features including fibrosis and inflammatory infiltration (10). The Notch signaling pathway contributes to chemoresistance in HCC through crosstalk with developmental signaling networks. Cancer Stem Cells (CSCs), a unique subpopulation of cancer cells, exhibit self-renewal capabilities, significant heterogeneity, and drug resistance, and are the principal contributors to tumor recurrence and metastasis (49). In liver cancer stem cells (LCSCs), Notch1 functions downstream of Wnt/β-catenin signaling, relying on Wnt activation to NICD, yet paradoxically establishes a negative feedback loop suppressing β-catenin/TCF activity—demonstrated by increased β-catenin activity upon Notch1 knockdown and reduced transcriptional output following NICD overexpression. This bidirectional crosstalk suggests that coordinated targeting of Notch and Wnt/β-catenin signaling networks may provide a novel therapeutic strategy to eliminate LCSCs and improve HCC treatment (50). The application of valproic acid (VPA) was shown to significantly inhibit the Notch/AKT signaling pathway, thereby restoring the cells’ sensitivity to sorafenib (11). Hang et al. reported that ZLDI-8, an inhibitor of ADAM17, effectively inhibits the cleavage and subsequent release of the NICD, thereby suppressing the transmission of the NOTCH signaling pathway. This inhibition leads to a downregulation of integrin β1 and β3 expression, ultimately restoring the sensitivity of HCC cells to sorafenib (12).

**Table 1:** Current status of chemicals that affect the angiogenic pathway of hepatocellular carcinoma.

Chemical	Signaling pathway	Role	Type of study	Reference
DAPT	NOTCH	γ-secretase inhibitor; blocks NICD release	preclinical study	(9)
Matrine	NOTCH	Promotes HOC differentiation into hepatocytes	preclinical study	(10)
Valproic acid	NOTCH	Restores the cells' sensitivity to sorafenib	preclinical study	(11)
ZLDI-8	NOTCH	NICD release blocker, restores sorafenib sensitivity	preclinical study	(12)
CB-103	NOTCH	NICD-CSL interaction blocker;inhibits downstream transcriptional activation	NCT03422679(Phase I)	(13)
LGK-974	Wnt/β-catenin	Wnt3A inhibitor; enhances HepG2 cell radiosensitivity	preclinical study	(14)
WAY-262611	Wnt/β-catenin	DKK1 inhibitor; suppresses Wnt/β-catenin signaling via GSK3β modulation; enhances sorafenib efficacy	preclinical study	(15)
PRI-724	Wnt/β-catenin	Selective CBP/β-catenin interaction disruptor; inhibits HCC proliferation and stemness	preclinical study	(16)
ICG-001	Wnt/β-catenin	Selective CBP/β-catenin interaction disruptor; inhibits HCC proliferation	Preclinical study	(17)
Trebananib	Ang/tie	Angiopoietin-neutralizing agent; normalizes tumor vasculature, reduces metastasis	NCT00872014(Phase II)	(18, 19)
BLU-554	FGF	FGFR4 inhibitor; synergizes with anti-PD-L1 to suppress metastasis via immune remodeling	NCT02508467(Phase I)	(20, 21)
FGF401	FGF	FGFR4 inhibitor; shows monotherapy efficacy and PD-1 combination potential	NCT02325739(Phase I/II)	(22)
Lenvatinib	FGF,VEGF	Multikinase inhibitor;iinduces anti-angiogenic and pro-apoptotic effects,enhances anti-PD-1 response	NCT01761266(Phase III)	(23, 24)
Deguelin	FGF	Suppresses angiogenesis and tumor-stroma interactions	preclinical study	(25)
Fangchinoline	HGF	Inhibits HCC proliferation and metastasis	preclinical study	(26)
DCN-PS	HGF	Competitive c-MET binder; inhibits invasion	preclinical study	(27)
CRI9	HGF	C-Met inhibitor;induces apoptosis in sorafenib-resistant HCC	preclinical study	(28)
AMG337	HGF	C-Met inhibitor;induces proliferation	preclinical study	(29)
H11	HGF	C-Met degrader; induces ubiquitination-dependent c-MET degradation to overcome drug resistance	preclinical study	(30)
Tepotinib	HGF	C-Met inhibitor;suppresses proliferation and metastatic	NCT01988493(Phase Ib/II )	(31)
Capmatinib	HGF	selective inhibitor of C-MET; induces angigenesis	NCT01737827(Phase II)	(32)
Tivantinib	HGF	C-Met inhibitor;suppresses proliferation	NCT01755767(Phase III)	(33)
Sorafenib	VEGF, FGF	Multikinase inhibitor;suppresses angiogenesis	NCT00105443(Phase III)	(34)
Bevacizumab	VEGF	Anti-VEGF monoclonal antibody; reducing tumor vascularization	NCT00162669(Phase II)	(35)
Ramucirumab	VEGF	VEGFR2inhibitor; suppresses angiogenesis	NCT02435433(Phase III)	(36)
Cabozantini	VEGF, HGF	Multikinase inhibitor; suppresses angiogenesis	NCT01908426(Phase III)	(37)
Apatinib	VEGF	VEGFR2inhibitor; blocks angiogenesis and EMT	NCT02329860(Phase III)	(38, 39)
LY294002	PI3K/AKT	PI3K inhibitor; enhances sorafenib sensitivity	preclinical study	(40)
DZW-310	PI3K/AKT	PI3Kα isoform-selective inhibitor; disrupts HIF-1α/VEGFA axis and tumor vascular remodeling	preclinical study	(41)
Ophiopogon	PI3K/AKT	PI3K/AKT inhibitor; suppresses proliferation, migration, and angiogenesis	preclinical study	(42)
ASP	PI3K/AKT	Downregulates HIF-1α/VEGF to suppress hypoxia-driven migration, invasion, and angiogenesis	preclinical study	(43)
Salvigenin	PI3K/AKT	PI3K/AKT/GSK-3β inhibitor; enhances 5-FU sensitivity by suppressing glycolysis and promoting apoptosis	preclinical study	(44)
Anhydroicaritin	PI3K/AKT	Natural flavonoid derived from Epimedium; suppress proliferation and metastasis	preclinical study	(45)
Rapamycin	PI3K/AKT	mTOR inhibitor; suppresses angiogenesis and proliferation	NCT00467194(Phase I)	(46)
Temsirolimus	PI3K/AKT	mTOR inhibitor; suppresses angiogenesis and proliferation	NCT00321594(Phase I/II)	(47)
Sirolimus	PI3K/AKT	mTOR inhibitor; suppresses angiogenesis, proliferation, metastasis	NCT00355862	(48)

Although NOTCH pathway inhibitors have demonstrated promising efficacy in preclinical models, their clinical translation faces significant challenges. In the HCC field, the sole ongoing clinical trial is NCT03422679—a non-randomized, open-label Phase I/II dose-escalation study evaluating the NOTCH inhibitor CB-103 in patients with advanced malignancies including HCC (13). Notably, dose-limiting toxicities of NOTCH inhibitors predominantly manifest as severe gastrointestinal adverse events, closely linked to the pathway’s essential roles in intestinal stem cell maintenance and epithelial barrier function (51, 52). These toxicities frequently lead to trial discontinuation or dose reduction, underscoring the imperative for tissue-selective delivery systems or rational polytherapy strategies.

## 3 Wnt/β-catenin pathway

The Wnt signaling pathway is important in the human body, regulating many important physiological processes in embryonic development and tissue homeostasis (53). In the canonical WNT signaling pathway, the WNT ligand engages with the corresponding Frizzled receptor and the co-receptor LRP5/6 on the cell surface. This interaction leads to the accumulation of β-catenin in the cytoplasm, which subsequently translocates to the nucleus. Within the nucleus, β-catenin interacts with the transcription factor TCF, thereby initiating the transcription of target genes and promoting various biological effects (**Figure 1**) (54, 55).

Previous studies have shown that targetin Porcupine O-acyltransferase (PORCN) or tankyrase (TNKS) may have some effects, such as the use of Wnt3A inhibitor LGK-974, which blocks Wnt signaling and prevents Nrf2 signaling and enhances the radiosensitivity of HepG2 cells (14). Sang Hyun et al. reported that the application of the DKK1 inhibitor WAY-262611 can suppress the PI3K/AKT and Wnt/β-catenin signaling pathways through the modulation of Glycogen synthase kinase 3 beta (GSK-3β) activity, thereby markedly augmenting the anti-tumor effectiveness of sorafenib (15). While therapeutic targeting of the Wnt/β-catenin pathway shows potential in HCC, its clinical translation faces dual biological constraints. First, the pathway’s indispensable roles in gastrointestinal homeostasis and epithelial regeneration render systemic inhibition prone to dose-limiting toxicities, including intestinal stem cell depletion and impaired wound healing as evidenced by clinical trial data (5). Second, genetic heterogeneity in HCC pathogenesis—particularly CTNNB1-activating mutations and AXIN functional loss—confers intrinsic resistance to upstream pathway inhibitors targeting PORCN, or TNKS. These mutations establish β-catenin signaling autonomy through distinct mechanisms: CTNNB1 mutants evade proteasomal degradation, while AXIN-deficient tumors bypass the β-catenin destruction complex (56). In this context, selective targeting of the CREB-binding protein (CBP)/β-catenin nuclear transcriptional complex holds critical therapeutic significance. PRI-724, a selective inhibitor of Wnt/β-catenin/CBP signaling, inhibits the proliferation of cultured HCC cells (57). Furthermore, nanoparticle-based delivery systems have shown significant potential for HCC therapy. A study demonstrated that niclosamide-loaded pluronic nanoparticles (NIC-NPs) enhanced antitumor efficacy in HCC-bearing rats compared to free niclosamide by prolonging drug release, restoring liver function, and amplifying Wnt/β-catenin/Notch pathway inhibition and apoptosis, while their negatively charged surface improved tumor targeting and safety through reduced off-target uptake, supporting nanoparticle-based strategies as a promising therapeutic avenue for HCC (16). In addition, Studies have shown that the combination of ICG-1 and anti-PD-1 antibody can promote the infiltration of DCs and CD8+ T cells within the TME, thereby enhancing immune cell activity and inhibiting tumor growth. Therefore, combining Wnt/β-catenin signaling pathway inhibitors with anti-PD-1 therapy may represent a promising treatment strategy for HCC patients (58).

Collectively, these findings establish a novel therapeutic paradigm for HCC by synergistically integrating β-catenin transcriptional inhibitors, nanoparticle-based drug delivery, and immune checkpoint blockade to overcome biological heterogeneity, enhance therapeutic precision, and mitigate systemic toxicity (**Table 1**).

## 4 Ang/Tie pathway

The Angiopoietin/Tie signaling pathway comprises four ligands, specifically ANG1 through ANG4. The Tie receptor, characterized by its tyrosine kinase activity, is extensively expressed in vascular endothelial cells. Angiopoietins form multimers via the SCD domain, subsequently binding to the Tie receptor. This interaction facilitates the autophosphorylation of Tie, thereby activating downstream signaling pathways that culminate in the transcription of specific target genes (**Figure 1**) (17). The Ang/Tie signaling pathway is integral to vascular remodeling, mural cell recruitment, and the maturation of the vasculature (59, 60).

Ang-1 and Ang-2 are considered the two most critical and extensively studied ligands within the angiopoietin family. Ang-1 primarily contributes to the maintenance of barrier function and homeostasis in vascular endothelial cells through its interaction with the Tie2 receptor. Conversely, Ang-2 generally acts as an antagonist to the Tie2 receptor, exhibiting variable functions contingent upon the levels of vascular endothelial growth factor (VEGF). In the absence of VEGF, Ang-2 may induce vascular regression, whereas in the presence of VEGF, it facilitates angiogenesis. Consequently, the Ang-1/Ang-2 ratio is of paramount importance in assessing the stability of the vascular system (61). The proliferative impact of Ang-2 on HCC is intricately associated with VEGF. The study conducted by Adriana et al. demonstrates that Ang-2 and VEGF synergistically augment the invasive capacity of HCC, with the underlying mechanism being closely linked to the induction of epithelial-mesenchymal transition (EMT). The concurrent targeting of Ang-2 and VEGF using Trebananib and Bevacizumab effectively inhibits the metastatic potential of the tumor (62). Furthermore, Ang-2 has the capability to activate the ERK-MSK signaling cascade, which subsequently induces the expression of downstream genes associated with drug resistance and anti-apoptosis, specifically Survivin and Ref-1. This molecular mechanism contributes to the resistance of HCC cells to the chemotherapeutic agent doxorubicin (18). Targeting the Angiopoietin/Tie2 signaling pathway has demonstrated promising efficacy in preclinical studies for the treatment of various solid tumors, including ovarian and gastrointestinal cancers (63). Nevertheless, research specifically focusing on HCC remains limited. In a study conducted by Kyriakos P et al., a notable reduction in alpha-fetoprotein (AFP) levels was observed in two HCC patients who maintained stable disease following treatment with Trebananib (64). However, when Ghassan K et al. used Trebananib in combination with sorafenib to treat HCC, compared with sorafenib alone, it did not show better treatment effects on HCC. The conflicting OS outcomes likely reflect methodological artifacts, particularly small sample size and sequential cohort accrual biasing baseline risk, rather than a true biological dose response (65). Therefore, further research is required to substantiate the efficacy of specifically targeting the Ang/Tie pathway in the treatment of HCC.

## 5 FGF pathway

Fibroblast growth factor(FGF) is a cytokine characterized by its multifaceted biological functions, influencing early embryonic development, tissue repair, metabolic processes, and a variety of physiological activities within the human body (19). The extensive FGF family comprises approximately 18 ligands, which interact with their primary receptors, FGFR1-4. This interaction initiates the activation of downstream signaling pathways, including the MAPK, PI3K/AKT, PKC, and STAT pathways, ultimately facilitating the expression of target genes (**Figure 1**).

The FGF/FGFR signaling pathway is aberrantly activated in numerous solid tumors, including ovarian, lung, and liver cancers, thereby facilitating tumor proliferation, metastasis, angiogenesis, and the development of drug resistance (66). Among FGF family members, FGF1, FGF2, FGF4, and FGF8 have demonstrated pro-angiogenic roles in various models, with FGF1 and FGF2 exhibiting particularly prominent effects. Notably, FGFs and VEGF exhibit compensatory interactions, such as FGF2 upregulating VEGFA expression to promote angiogenesis. Anti-VEGF therapies may induce compensatory activation of FGF pathways, leading to drug resistance. Studies suggest that dual targeting of these pathways may yield superior antitumor efficacy (67). Current research predominantly focuses on FGFR4 in HCC, where it shows significantly higher overexpression compared to FGFR1-3. FGFR4 emerges as the predominantly overexpressed FGFR isoform in HCC, with minimal upregulation observed in FGFR1/2. Notably, FGFR4-selective inhibitors circumvent hyperphosphatemia caused by FGFR1/3 inhibition, demonstrating improved safety profiles. These attributes position FGFR4 as a promising therapeutic target in HCC (68). The FGF19/FGFR4 axis contributes to HCC resistance to sorafenib by inhibiting ROS-related apoptosis induced by the drug (69). The signaling pathway facilitates the expression of ETV4, which subsequently enhances the expression of death-ligand 1 (PD-L1) and CCL2. This upregulation leads to increased infiltration of tumor-associated macrophage (TAM) and myeloid-derived suppressor cells (MDSC), while concurrently inhibiting the accumulation of CD8+ T cells, thereby promoting an immunosuppressive environment. In addition,ETV4 is capable of enhancing the transcription and translation of FGFR4, thereby facilitating the formation of the FGFR4-ERK1/2-ELK1 positive feedback pathway. In this study, the combined application of the FGFR4 inhibitor BLU-554 and anti-PD-L1 therapy was shown to significantly suppress HCC metastasis (70, 71). A clinical study demonstrates that FGF401 (roblitinib) is a highly efficient and specific inhibitor targeting FGFR4. It exhibits a favorable therapeutic effect when used either as a monotherapy for HCC or in combination with a PD-1 inhibitor (20). Lenvatinib has been demonstrated to decrease PD-L1 expression in HCC and inhibit the differentiation of regulatory regulatory T cells (Tregs) through the blockade of FGFR-4. These mechanisms contribute to an augmented therapeutic efficacy of anti-PD-1 inhibitors (21).

Overall, targeting the FGF/FGFR axis—particularly FGFR4—represents a promising therapeutic strategy for HCC. Emerging evidence underscores the necessity of combination approaches, including co-targeting FGFR4 with other pathways or immune checkpoint inhibitors, to overcome compensatory mechanisms and enhance clinical efficacy (**Table 1**).

## 6 HGF pathway

Hepatocyte Growth Factor(HGF)is a multifunctional cytokine that serves as a mitogen for various epithelial cells. The receptor for HGF is C-MET, a transmembrane receptor tyrosine kinase. Upon specific binding of HGF to C-MET, phosphorylation of the tyrosine residues on C-MET occurs, subsequently activating multiple downstream signaling pathways via SRC. This activation leads to the expression of target genes, influencing tissue regeneration, ameliorating fibrosis and inflammatory responses, and modulating the expression of vascular-related growth factors, such as VEGF (**Figure 1**) (22, 23, 72).

Aberrant expression of HGF often exerts tumor-promoting effects (73). HGF has been demonstrated to drive the initiation and progression of multiple cancers, including HCC, with its mechanisms extensively studied. Under physiological conditions, the HGF/c-MET signaling pathway is tightly regulated and maintained at stable levels. However, in HCC, this pathway becomes abnormally activated. Compared to normal liver tissues, C-MET mRNA levels are significantly elevated in HCC tissues (74). A substantial body of preclinical studies has validated the feasibility of targeting the HGF/c-MET pathway for HCC treatment. For instance, Ming et al. showed that Deguelin suppresses VEGF secretion by targeting the HGF/c-MET pathway, thereby inhibiting angiogenesis in HCC (75). Similarly, Fangchinoline negatively regulates the c-MET/HGF axis and its associated downstream signaling pathways, leading to HCC proliferation inhibition (76). Yong et al. demonstrated that DCN-derived peptides(DCN-PS) competitively bind to c-MET, blocking the HGF/c-MET signaling pathway and impeding HCC progression (25). CRI9, a novel synthetic compound, inhibits c-MET phosphorylation, thereby suppressing the downstream PI3K/AKT/mTOR pathway. This mechanism has shown significant anti-tumor effects in murine models (26). AMG337 has been shown to potently inhibit the proliferation of HCC cells with elevated c-MET expression (27). The HGF pathway regulates HCC resistance through multiple mechanisms. For example, it reduces HCC sensitivity to sorafenib by modulating the AKT/ERK1/2-ERG1 axis (58). Additionally, c-MET activates the MAPK signaling cascade, upregulating NF-κB mediated PD-L1 expression, thereby enhancing immune evasion and drug resistance (28). H11, a novel c-MET degrader, induces c-MET ubiquitination-proteasome degradation and demonstrates anti-tumor activity while overcoming drug resistance (29).

Despite promising preclinical results, clinical trials of HGF/c-MET inhibitors have yielded mixed outcomes. In a Phase 1b/2 trial involving systemic anticancer treatment-naive Asian patients with MET-overexpressing advanced hepatocellular carcinoma, tepotinib demonstrated improved independently assessed time to progression and a lower rate of grade ≥3 treatment-related adverse events compared to sorafenib, supporting its potential efficacy and tolerability in this population (77). Capmatinib, a highly selective inhibitor of C-MET, demonstrated promising anti-tumor efficacy in a phase II clinical trial targeting HCC characterized by high C-MET expression levels (30). However, its lack of FDA approval may stem from intrinsic resistance mechanisms linked to immune adaptation. DeAzevedo et al. further demonstrated that type I MET inhibitors, including capmatinib, induce compensatory PD-L1 elevation in HCC models, rendering tumors resistant to MET-targeted therapy but sensitized to PD-1 blockade. This mechanistic synergy underscores the potential of combining capmatinib with anti-PD-1 agents to simultaneously disrupt MET-driven oncogenesis and immune evasion (31). Tivantinib, a selective c-MET inhibitor, failed to meet primary endpoints in a Phase III trial, showing no significant improvement in overall survival (OS) or progression free survival (PFS) compared to placebo. Its high toxicity profile, including ascites, anemia, abdominal pain, and neutropenia, may reflect the physiological roles of HGF/c-MET in multiple organs (32).

Overall, the HGF/c-MET pathway remains a valid therapeutic target for HCC. The limited success of clinical trials is attributed to inconsistent detection standards, unresolved molecular heterogeneity, and underestimated immune evasion mechanisms. Future directions include optimizing diagnostic techniques, stratifying patients for targeted therapy, and exploring combination immunotherapy. Further prospective studies are warranted to validate c-MET as a therapeutic target (**Table 1**).

## 7 VEGF pathway

The VEGF signaling pathway plays a pivotal role in normal vascular development and growth, as well as in tumor angiogenesis. The VEGF family comprises several members, including VEGF-A, VEGF-B, VEGF-C, VEGF-D, and PGF. The primary VEGF receptors include VEGFR1, VEGFR2, and VEGFR3, each serving distinct functional roles. VEGFR2 is principally responsible for mediating angiogenesis, while VEGFR3 predominantly regulates lymphangiogenesis (77, 78). Upon ligand-induced dimerization, the activated receptor triggers a sequence of downstream signaling pathways, including RAS/MAPK, SRC, and PI3K/AKT, culminating in the activation of relevant angiogenic gene expression (**Figure 1**) (79).

VEGF, as the most critical factor in tumor angiogenesis, has become the primary therapeutic target for antiangiogenic strategies. Current antiangiogenic multi-kinase inhibitor, revolutionized HCC treatment by significantly improving median OS in advanced disease, as demonstrated in the SHARP trial. This marked a paradigm shift in liver cancer treatment, transitioning from traditional approaches (surgery, locoregional therapies) to targeted therapies. Bevacizumab inhibits tumor growth by neutralizing VEGF-VEGFR interactions (80, 81). Ramucirumab, a VEGFR2-targeted monoclonal antibody, showed efficacy in AFP-high HCC patients (82). Furthermore, agents such as lenvatinib and cabozantinib have shown efficacy in HCC management (34, 35). These successes underscore the pivotal role of VEGFR inhibition in HCC therapy. Inhibition of the VEGF/VEGFR axis confers multiple therapeutic benefits, including enhanced efficacy of ICIs. Antiangiogenic agents transiently improve vascular architecture by enhancing pericyte coverage and reducing vascular leakage, thereby increasing tumor perfusion and oxygenation to create a vascular normalization window. This window alleviates hypoxia, enhances immune cell infiltration, and improves drug delivery efficiency (36). Clinical studies demonstrate that combining antiangiogenic TKIs with ICIs during this window synergistically enhances antitumor efficacy. For instance, the combination of bevacizumab and atezolizumabhas shown promising survival benefits in advanced HCC, as evidenced by recent clinical trials (24).

The VEGF/VEGFR signaling axis remains central to antiangiogenic therapy in HCC, supported by decades of clinical success and emerging synergies with immunotherapy (83, 84) (**Table 1**). While challenges such as tumor heterogeneity and resistance persist, ongoing research into novel agents and combination strategies continues to refine this paradigm, solidifying the pathway’s enduring relevance in HCC management.

## 8 PI3K/AKT pathway

PI3K is a heterodimer composed of a catalytic subunit, p110, and a regulatory subunit, p85 [98]. AKT, also referred to as protein kinase B, functions as a serine/threonine kinase. The PI3K/AKT signaling pathway frequently serves as a mediator, modulating diverse cellular processes including proliferation, metabolism, and angiogenesis, upon activation by various upstream signals. (**Figure 1**) (37).

The PI3K/AKT pathway drives oncogenesis through multi-layered regulatory mechanisms (**Table 2**). A key example is AKT-mediated phosphorylation of HIF-1α at serine/threonine residues, which prevents its VHL-dependent ubiquitination and degradation. This stabilizes HIF-1α under hypoxic conditions, enabling its accumulation and subsequent transcriptional activation of VEGFA to induce angiogenesis (89). Notably, HIF-1α orchestrates both angiogenic and metabolic reprogramming in hypoxia. Beyond activating VEGFA, HIF-1α upregulates glycolytic enzymes such as GLUT1 and LDHA, forcing cancer cells to adopt the Warburg effect for energy production. This metabolic shift enhances glucose uptake and lactate secretion, supporting tumor proliferation even in oxygen-deprived microenvironments. Critically, the accumulated lactate further stabilizes HIF-1α by inhibiting prolyl hydroxylase (PHD) activity, thereby creating a self-reinforcing feedforward loop that perpetuates glycolysis and angiogenesis. HIF-1α overexpression also enhances tumor aggressiveness by promoting EMT, facilitating genetic instability, suppressing apoptosis, and exacerbating resistance to chemotherapy and radiotherapy (90, 91). In addition to HIF-1α regulation, PI3K/AKT signaling activates NF-κB, which transcriptionally upregulates VEGF expression, amplifying angiogenic signaling (38). Concurrently, the PI3K/AKT axis modulates angiogenesis via the eNOS/NO pathway, where AKT phosphorylates and activates endothelial nitric oxide synthase (eNOS) to boost nitric oxide production, thereby enhancing angiogenesis (39). Moreover, crosstalk between PI3K/AKT and Wnt/β-catenin pathways contributes to malignant progression. PI3K/AKT phosphorylates GSK-3β at Ser9, inhibiting its kinase activity and preventing β-catenin degradation (92). This stabilization of β-catenin activates Wnt signaling, which drives cancer stem cell proliferation and confers therapeutic resistance. Therefore, the development of inhibitors and combination therapies targeting this pathway has become a research focus.

**Table 2:** PI3K/AKT-mediated signaling crosstalk with VEGF and WNT/β-catenin pathways in cancer progression.

Interaction	Mechanism	Function	Reference
PI3K/AKT&VEGF	VEGF/VEGFR/PI3K/AKT	forming a positive feedback loop that promotes angiogenesis EMT, metastasis, and drug resistance	(80, 85)
	PI3K/AKT/HIF-1/VEGF		
	PI3K/AKT/NF-KappaB/VEGF	Angiogenesis, inflammation	(86)
	VEGF/PI3K/AKT/eNOS/NO	angiogenesis	(87)
PI3K/AKT&WNT/β-catenin	PI3K/AKT/GSK-3β/β-catenin	angiogenesis,proliferationmetastasis,metastasis,drug resistance,EMT	(57, 88)

Preclinical studies demonstrate that the PI3K inhibitor LY294002 suppresses the AKT/GSK-3β signaling pathway, enhancing the VEGFR2-targeting effect of sorafenib. This combination promotes vascular normalization and reverses drug resistance (93). DZW-310, a novel PI3K inhibitor, disrupts the HIF-1α/VEGFA axis by inhibiting the PI3K/AKT pathway, thereby attenuating angiogenesis in HCC (94). Additionally, several plant-derived components exhibit inhibitory effects on the PI3K/AKT pathway. Ophiopogonin-B suppresses the PI3K/AKT pathway via downregulation of protein tyrosine phosphatase 1B (PTP1B) while activating the AMP-activated protein kinase (AMPK) pathway, leading to inhibition of HCC cell proliferation, migration, and angiogenic capabilities (95). ASP, a significant phytoextract, downregulates HIF-1α/VEGF expression by inhibiting PI3K and MAPK signaling pathways, suppressing hypoxia-induced migration, invasion, and angiogenesis in HCC cells (85). Salvigenin impedes aerobic glycolysis and enhances sensitivity to 5-fluorouracil(5-FU) in HCC cells by inhibiting the PI3K/AKT/GSK-3β pathway, restraining tumor growth in nude mice and promoting apoptosis (86). Anhydroicaritin(AHI), derived from traditional Chinese medicine, has been shown *in vitro* to effectively inhibit the PI3K/AKT pathway, suppressing HCC cell proliferation and metastasis (87).

In a Phase I study of rapamycin plus bevacizumab for advanced HCC, early efficacy was demonstrated, with 1 complete response (lasting 4.5 months) and 2 partial responses observed among 20 evaluable patients, alongside manageable toxicity (88). For temsirolimus, an mTOR inhibitor, a Phase I/II trial in unresectable advanced HCC did not meet the primary endpoint of PFS (95). The SiLVER trial investigated sirolimus for preventing HCC recurrence after liver transplantation, but the primary endpoint of improved disease-free survival (DFS) was not achieved. However, subsequent multivariate analysis revealed that everolimus improved outcomes in a subgroup with high tumor activity indicated by AFP levels, advocating its use in this population (41). A Phase II study of temsirolimus combined with sorafenib in advanced HCC showed favorable safety and improved OS, though outcomes fell short of expectations (42).

As a pivotal downstream signaling node, PI3K/AKT plays critical roles in HCC angiogenesis, proliferation, and other oncogenic processes. Preclinical studies targeting PI3K/AKT show robust antitumor effects, though clinical trials have yielded mixed results. Future strategies may require combination therapies and biomarker-guided approaches to optimize PI3K/AKT-targeted interventions in HCC (**Table 1**).

## 9 Biomarker for personalized anti-angiogenic therapy

The molecular and phenotypic heterogeneity of HCC, evidenced by tumor microenvironmental variations and divergent angiogenic pathway activation patterns, fundamentally limits the efficacy of anti-angiogenic regimens, underscoring the urgent need to identify predictive biomarkers.

early reductions in circulating VEGF levels predict favorable responses to sorafenib, while sustained elevation correlates with treatment resistance and adverse prognosis in sorafenib-treated patients (43, 44). The serum concentration of Ang-2 may serve as a biomarker for evaluating the therapeutic efficacy of systemic treatment agents, such as sorafenib and regorafenib, in patients with advanced HCC (45, 46). Notably, baseline Ang-2/VEGF synergy has been validated as a personalized prognostic tool for lenvatinib-treated patients, where combined low Ang-2/high VEGF profiles portend accelerated Child-Pugh deterioration (47). Elevated serum DKK-1, a Wnt signaling modulator, serves as a prognostic biomarker in HCC by driving tumor stemness, angiogenesis, and invasion (48). Elevated Notch1 and Notch4 expression independently predicts shorter recurrence-free and disease-specific survival in HCC patients post-curative resection, as demonstrated by Soomin Ahn et al., positioning these receptors as dual prognostic biomarkers and potential therapeutic targets for anti-angiogenic strategies (96). Studies by Xiang et al. demonstrated a potential association between MET expression and HCC patient responses to sorafenib therapy, revealing that HCC patients with elevated phosphorylated MET (p-MET) levels exhibited resistance to adjuvant sorafenib treatment, suggesting that MET activation in HCC may serve as a promising predictive biomarker for therapeutic response (97). High FGFR4 expression and tumor-infiltrating Tregs synergistically predict enhanced therapeutic response to lenvatinib plus anti-PD-1 combination therapy in HCC, positioning these biomarkers for clinical stratification of patients likely to benefit from this regimen (21).

Collectively, these findings underscore the imperative to develop multi-dimensional biomarker panels that address HCC’s biological complexity while enabling real-time therapeutic adaptation.

## 10 Tumor microenvironment and therapeutic implications in HCC

Tumor neovasculature sustains tumor growth by delivering oxygen and nutrients while removing metabolic waste products. However, structural abnormalities (e.g., high permeability and poor perfusion) create a hypoxic and acidic tumor microenvironment (TME), which stimulates the secretion of pro-angiogenic factors (e.g., VEGF and Ang-2). This process further establishes a self-perpetuating “angiogenesis-hypoxia-immunosuppression” vicious cycle (98). The TME comprises a dynamic network of tumor cells, endothelial cells, immune cells (e.g., TAMs, Tregs), cancer-associated fibroblasts (CAFs), and extracellular matrix (ECM), along with soluble factors such as cytokines and growth factors. These components engage in bidirectional crosstalk to promote tumorigenesis. For instance, CAFs secrete VEGF, and matrix metalloproteinases (MMPs) to remodel the ECM, facilitating angiogenesis and tumor invasion. Hypoxic polarization of TAMs toward the immunosuppressive M2 phenotype enhances their secretion of PD-L1, arginase-1 (ARG1), and interleukin-10 (IL-10), thereby suppressing CD8^+^ T-cell function while reinforcing angiogenesis via VEGF-A and fibroblast growth factor-2 (FGF-2) (99, 100). Notch signaling increases the secretion of inflammatory factors interleukin-6 (IL-6) and (Inducible Nitric Oxide Synthase)iNOS, decreases the release of IL-10, and polarizes macrophages toward M1 (101). Concurrently, Wnt/β-catenin activation in tumor cells triggers paracrine Wnt ligand secretion, polarizing macrophages toward the M2 phenotype and establishing a protumor immunoprivileged niche (54). Moreover, the HGF/c-MET and FGF19/FGFR4 axes synergize via the ERK1/2-ETV4 axis to upregulate PD-L1 and CCL2 expression in TAMs and MDSCs, suppressing CD8^+^ T-cell activity and driving HCC metastasis (70). This interconnected TME network not only fosters tumor progression but also induces resistance to antiangiogenic therapies, underscoring the imperative for combinatorial targeting of TME components in HCC therapy.

While antiangiogenic drugs disrupt tumor blood supply, their efficacy is often limited by compensatory signaling pathways and immunosuppressive feedback loops mediated by CAFs and TAMs. Therefore, integrating TME-modulating therapies may offer multifaceted solutions to overcome drug resistance and improve outcomes in these aggressive malignancies. For instance, FAP-targeted vaccines have demonstrated antitumor activity in preclinical models, modulating the immunosuppressive microenvironment while reducing tumor growth and angiogenesis (90). The integration of anti-angiogenic agents with immune checkpoint inhibitors has emerged as a transformative strategy in advanced HCC management. The landmark IMbrave150 trial demonstrated that combining the anti-PD-L1 agent atezolizumab with the anti-VEGF monoclonal antibody bevacizumab significantly improved median OS to 19.2 months compared to 13.4 months achieved by sorafenib monotherapy, establishing this dual-mechanism regimen as the first-line standard of care. This synergy arises from VEGF blockade normalizing tumor vasculature while PD-L1 inhibition reverses T-cell exhaustion, creating a permissive microenvironment for immune-mediated tumor control. Subsequent combinations have shown comparable efficacy, including the anti-PD-1 agent sintilimab paired with bevacizumab, which achieved a median OS of 20.2 months and a 21% objective response rate in the ORIENT-32 trial (102). Phase II-tested combination of the anti-PD-1 agent camrelizumab with the VEGFR2 inhibitor apatinib, yielding a 20.1-month median OS (103). The remarkable clinical successes of angiogenesis-immune checkpoint combinations represent more than incremental progress-they herald a new era of rationally designed multi-mechanistic therapies for HCC.

## 11 Conclusions and perspectives

The interplay of multiple signaling pathways drives HCC progression by promoting angiogenesis, immune evasion, and therapeutic resistance. However, monotherapies face significant limitations: compensatory mechanisms rapidly reactivate angiogenesis, while pathway-specific inhibitors blockers cause severe toxicities that restrict clinical application. These challenges highlight the adaptive resilience of HCC and underscore the urgent need for innovative strategies. Nanoparticle-based delivery systems emerge as a transformative solution, enabling precise drug delivery to overcome toxicity barriers and enhance therapeutic efficacy. The TME drives resistance by creating a self-sustaining cycle where angiogenesis and immune evasion mutually reinforce each other. This interplay underscores the clinical success of combining anti-angiogenic TKIs with ICIs, as demonstrated by the survival benefit of atezolizumab-bevacizumab in advanced HCC.in addition, Integrating TME-modulating agents may provide a multifaceted solution to overcome resistance and improve outcomes in this lethal malignancy.

Future therapeutic strategies should prioritize the utilization of predictive biomarkers to mitigate efficacy limitations caused by the molecular and phenotypic heterogeneity of HCC, while emphasizing rational polytherapy approaches—including multi-targeted therapies, TKI-ICI combinations, TME-modulating agents, and nanoparticle-enhanced drug delivery systems. These strategies aim to simultaneously disrupt angiogenesis, restore immune surveillance, improve therapeutic efficacy and safety profiles, and overcome compensatory signaling pathways in HCC.
