# Supplementary material for: Aberrant angiogenic signaling in HCC: therapeutic targeting and drug resistance
Source: Front Oncol. 2025 Jun 18;15:1595195. doi: 10.3389/fonc.2025.1595195 (PMC12213894; doi:10.3389/fonc.2025.1595195)
Supplement: Supplementary file 1 [file DataSheet1.docx]

**Table 1**. Current status of Chemicals that affect the angiogenic pathway of hepatocellular carcinoma

| Chemical | Signaling Pathway | Role | Type of study | Reference |
| --- | --- | --- | --- | --- |
| DAPT | NOTCH | γ-secretase inhibitor; blocks NICD release | preclinical study | ([9](#_ENREF_9)) |
| Matrine | NOTCH | Promotes HOC differentiation into hepatocytes | preclinical study | ([10](#_ENREF_10)) |
| Valproic acid | NOTCH | Restores the cells' sensitivity to sorafenib | preclinical study | ([13](#_ENREF_13)) |
| ZLDI-8 | NOTCH | NICD release blocker, restores sorafenib sensitivity | preclinical study | ([14](#_ENREF_14)) |
| CB-103 | NOTCH | NICD-CSL interaction blocker;inhibits downstream transcriptional activation | NCT03422679(Phase Ⅰ) | ([15](#_ENREF_15)) |
| LGK-974 | Wnt/β-catenin | Wnt3A inhibitor; enhances HepG2 cell radiosensitivity | preclinical study | ([21](#_ENREF_21)) |
| WAY-262611 | Wnt/β-catenin | DKK1 inhibitor; suppresses Wnt/β-catenin signaling via GSK3β modulation; enhances sorafenib efficacy | preclinical study | ([22](#_ENREF_22)) |
| PRI-724 | Wnt/β-catenin | Selective CBP/β-catenin interaction disruptor; inhibits HCC proliferation and stemness | preclinical study | ([25](#_ENREF_25)) |
| ICG-001 | Wnt/β-catenin | Selective CBP/β-catenin interaction disruptor; inhibits HCC proliferation | Preclinical study | ([27](#_ENREF_27)) |
| Trebananib | Ang/tie | Angiopoietin-neutralizing agent; normalizes tumor vasculature, reduces metastasis | NCT00872014(Phase Ⅱ) | ([32](#_ENREF_32), [36](#_ENREF_36)) |
| BLU-554 | FGF | FGFR4 inhibitor; synergizes with anti-PD-L1 to suppress metastasis via immune remodeling | NCT02508467(Phase Ⅰ) | ([42](#_ENREF_42), [43](#_ENREF_43)) |
| FGF401 | FGF | FGFR4 inhibitor; shows monotherapy efficacy and PD-1 combination potential | NCT02325739(Phase Ⅰ/Ⅱ) | ([44](#_ENREF_44)) |
| Lenvatinib | FGF,VEGF | Multikinase inhibitor;iinduces anti-angiogenic and pro-apoptotic effects,enhances anti-PD-1 response | NCT01761266(Phase Ⅲ) | ([45](#_ENREF_45), [69](#_ENREF_69)) |
| Deguelin | FGF | Suppresses angiogenesis and tumor-stroma interactions | preclinical study | ([51](#_ENREF_51)) |
| Fangchinoline | HGF | Inhibits HCC proliferation and metastasis | preclinical study | ([52](#_ENREF_52)) |
| DCN-PS | HGF | Competitive c-MET binder; inhibits invasion | preclinical study | ([53](#_ENREF_53)) |
| CRI9 | HGF | C-Met inhibitor;induces apoptosis in sorafenib-resistant HCC | preclinical study | ([54](#_ENREF_54)) |
| AMG337 | HGF | C-Met inhibitor;induces proliferation | preclinical study | ([55](#_ENREF_55)) |
| H11 | HGF | C-Met degrader; induces ubiquitination-dependent c-MET degradation to overcome drug resistance | preclinical study | ([58](#_ENREF_58)) |
| Tepotinib | HGF | C-Met inhibitor;suppresses proliferation and metastatic | NCT01988493(Phase Ⅰb/Ⅱ ) | ([59](#_ENREF_59)) |
| Capmatinib | HGF | selective inhibitor of C-MET; induces angigenesis | NCT01737827(Phase Ⅱ) | ([60](#_ENREF_60)) |
| Tivantinib | HGF | C-Met inhibitor;suppresses proliferation | NCT01755767(Phase Ⅲ) | ([62](#_ENREF_62)) |
| Sorafenib | VEGF, FGF | Multikinase inhibitor;suppresses angiogenesis | NCT00105443(Phase Ⅲ) | ([66](#_ENREF_66)) |
| Bevacizumab | VEGF | Anti-VEGF monoclonal antibody; reducing tumor vascularization | NCT00162669(Phase Ⅱ) | ([67](#_ENREF_67)) |
| Ramucirumab | VEGF | VEGFR2inhibitor; suppresses angiogenesis | [NCT02435433](http://clinicaltrials.gov/show/NCT02435433)(Phase Ⅲ) | ([68](#_ENREF_68)) |
| Cabozantini | VEGF, HGF | Multikinase inhibitor; suppresses angiogenesis | NCT01908426(Phase Ⅲ) | ([70](#_ENREF_70)) |
| Apatinib | VEGF | VEGFR2inhibitor; blocks angiogenesis and EMT | NCT02329860(Phase Ⅲ) | ([71](#_ENREF_71), [72](#_ENREF_72)) |
| LY294002 | PI3K/AKT | PI3K inhibitor; enhances sorafenib sensitivity | preclinical study | ([82](#_ENREF_82)) |
| DZW-310 | PI3K/AKT | PI3Kα isoform-selective inhibitor; disrupts HIF-1α/VEGFA axis and tumor vascular remodeling | preclinical study | ([83](#_ENREF_83)) |
| Ophiopogon | PI3K/AKT | PI3K/AKT inhibitor; suppresses proliferation, migration, and angiogenesis | preclinical study | ([84](#_ENREF_84)) |
| ASP | PI3K/AKT | Downregulates HIF-1α/VEGF to suppress hypoxia-driven migration, invasion, and angiogenesis | preclinical study | ([85](#_ENREF_85)) |
| Salvigenin | PI3K/AKT | PI3K/AKT/GSK-3β inhibitor; enhances 5-FU sensitivity by suppressing glycolysis and promoting apoptosis | preclinical study | ([86](#_ENREF_86)) |
| Anhydroicaritin | PI3K/AKT | Natural flavonoid derived from Epimedium; suppress proliferation and metastasis | preclinical study | ([87](#_ENREF_87)) |
| Rapamycin | PI3K/AKT | mTOR inhibitor; suppresses angiogenesis and proliferation | [NCT00467194](http://clinicaltrials.gov/show/NCT00467194)(Phase Ⅰ) | ([88](#_ENREF_88)) |
| Temsirolimus | PI3K/AKT | mTOR inhibitor; suppresses angiogenesis and proliferation | [NCT00321594](http://clinicaltrials.gov/show/NCT00321594)(Phase Ⅰ/Ⅱ) | ([89](#_ENREF_89)) |
| Sirolimus | PI3K/AKT | mTOR inhibitor; suppresses angiogenesis, proliferation, metastasis | [NCT00355862](http://clinicaltrials.gov/show/NCT00355862) | ([90](#_ENREF_90)) |

**Table 2. PI3K/AKT-mediated signaling crosstalk with VEGF and WNT/β-catenin pathways in cancer progression**

| Interaction | Mechanism | Function | Reference |
| --- | --- | --- | --- |
| PI3K/AKT&VEGF | VEGF/VEGFR/PI3K/AKT | forming a positive feedback loop that promotes angiogenesis EMT, metastasis, and drug resistance | ([64](#_ENREF_64), [78](#_ENREF_78)) |
|  | PI3K/AKT/HIF-1/VEGF |  |  |
|  | PI3K/AKT/NF-KappaB/VEGF | angiogenesis，inflammation | ([79](#_ENREF_79)) |
|  | VEGF/PI3K/AKT/eNOS/NO | angiogenesis | ([80](#_ENREF_80)) |
| PI3K/AKT&WNT/β-catenin | PI3K/AKT/GSK-3β/β-catenin | angiogenesis,proliferationmetastasis,metastasis,drug resistance,EMT | ([24](#_ENREF_24), [81](#_ENREF_81)) |
